# The global, regional, and national disease burden of breast cancer attributable to tobacco from 1990 to 2019: a global burden of disease study

**DOI:** 10.1186/s12889-023-17405-w

**Published:** 2024-01-06

**Authors:** Qiusheng Guo, Yunyan Lu, Weiguo Liu, Gaochen Lan, Tian Lan

**Affiliations:** 1 Department of Medical Oncology, Affiliated Jinhua Hospital, Zhejiang University School of Medicine, Jinhua, Zhejiang People’s Republic of China; 2 Department of Cardiology, The First People’s Hospital of Xiaoshan District, Xiaoshan Affiliated Hospital of Wenzhou Medical University, Hangzhou, Zhejiang People’s Republic of China; 3 Department of Oncology, The People’s Hospital of Jiangshan, Quzhou, Zhejiang People’s Republic of China; 4 Department of Oncology, The Second Affiliated Hospital of Fujian Medical University, Quanzhou, Fujian People’s Republic of China; 5 Department of Breast Surgery, Hangzhou TCM Hospital Affiliated to Zhejiang Chinese Medical University, Hangzhou Hospital of Traditional Chinese Medicine, Hangzhou, Zhejiang People’s Republic of China

**Keywords:** Breast cancer, Tobacco, Global burden of disease study

## Abstract

**Objective:**

Tobacco has been identified as a significant contributory element to the development of breast cancer. Our objective was to evaluate the spatiotemporal trends of tobacco-related breast cancer at the global, regional, and national scales during 1990–2019.

**Methods:**

We extracted data on mortality, disability adjusted of life years (DALYs), age-standardized mortality rate (ASMR), and age-standardized DALYs rate (ASDR) from the Global Burden of Disease (GBD) study 2019. Estimated annual percentage change (EAPC) was computed to assess the temporal change in ASDR and ASMR.

**Results:**

In 2019, the deaths and DALYs attributed to tobacco-related breast cancer were estimated to be 35,439 (95% UI: 22,179–48,119) and 1,060,590 (95% UI: 622,550–1,462,580), respectively. These figures accounted for 5.1% and 5.2% of the total burden of breast cancer. ASMR and ASDR increased in low SDI regions, remained stable in low-middle and middle SDI regions and declined in high and high-middle SDI regions. The burden of breast cancer attributable to tobacco varied notably among regions and nations. Oceania, Southern Latin America, and Central Europe were the GBD regions with the highest number of ASMR and DALYs. There was a positive relationship between age-standardized rate and SDI value in 2019 across 204 nations or territories. A negative association was observed between the EAPC in ASMR or ASDR and the human development index (HDI) in 2019 (R = -0.55, *p* < 0.01 for ASMR; R = -0.56, *p* < 0.01 for ASDR).

**Conclusion:**

Tobacco is one important and modifiable risk factor for breast cancer. The heterogeneity in both the spatial and temporal distribution can be attributed to factors such as aging, population growth, and SDI. These findings substantiate the necessity of expediting the enforcement of tobacco-free legislation in order to safeguard populations from the detrimental effects of tobacco.

**Supplementary Information:**

The online version contains supplementary material available at 10.1186/s12889-023-17405-w.

## Introduction

Breast cancer is the most frequently diagnosed cancer, accounting for 24.2% of all cancer cases worldwide among females [1]. It is recognized as the primary factor for cancer-related mortality among women [2, 3]. Various lifestyle risk factors contribute to the development of breast cancer, including consumption of alcohol and tobacco, obesity, and low physical activity [4]. Tobacco is acknowledged as a prominent risk factor contributing to approximately 200 million deaths and imposing economic costs amounting to $1 trillion over the three decades [5, 6]. Tobacco could also consistently release a substantial number of carcinogens, leading to millions of annual cancer-related deaths [7]. It has been reported that tobacco is closely linked to a modest yet significantly higher risk of breast cancer [8].

Thus far, numerous studies have been conducted to examine the global trends of breast cancer [9, 10]. There is a positive association between long-term smoking and the breast cancer risk [11, 12]. However, the epidemiological patterns and trends of tobacco-associated breast cancer remain unknown on a national, regional, and global scale. To enhance the formulation of health policies and lifestyle guidelines, it is worthwhile to consolidate and compare various metrics and trends pertaining to tobacco-related breast cancer from multiple perspectives. In this study, we assess the spatial and temporal progression of female breast cancer attributed to tobacco using data from the Global Burden of Disease (GBD) 2019 study.

## Methods

### Data sources

The GBD 2019 study, a collaborative multinational research effort, provided estimations of the burden of 329 diseases across 204 countries or territories, 21 regions, and 7 super-regions for the period of 1990–2019 [13]. The risk factors encompassed metabolic, occupational, environmental, and behavioral factors, including tobacco use. The data regarding the numbers and age-standardized rates (ASR) of tobacco-related breast cancer death and disability adjusted of life years (DALYs) from 1990 to 2019, categorized by age, region, country, were extracted from the GBD 2019 study using the online Global Health Data Exchange query tool (GHDx, http://ghdx.healthdata.org/gbd-results-tool). The information on human development index (HDI) among nations was obtained from the United Nations Human Development Report (http://hdr.undp.org/en/data).

### Estimated breast cancer burden due to tobacco

In GBD 2019 study, tobacco use encompassed multiple modalities, including current or past usage of any smoked tobacco product, current use of any chewing tobacco product, and the average daily exposure to air particulate matter from second-hand smoke [14].

Mortality data were estimated using a combination of high-quality cancer registered incidence and modeled mortality-to-incidence ratio. This estimation involved the application of spatiotemporal Gaussian process modeling techniques. DALYs were represented by the sum of years lived with disability (YLDs) and years of life lost (YLLs). One DALY was equivalent to one healthy year lost. ASRs, such as age-standardized mortality rate (ASMR) and age-standardized DALYs rate (ASDR), were utilized usually to compare the disease burden while accounting for variations in age structures, which were more accurate epidemiological assessments.

The individuals were classified into four distinct age groups (25–40, 40–55, 55–70, and 70 +) to assess the distribution of the disease across different age ranges. The socio-demographic index (SDI) was calculated according to national income per capita, total fertility rate among individuals younger than 25, and average schooling years among persons older than 15 [13]. Countries and territories were categorized into five subgroups based on SDI values, namely: low, low-medium, medium, high-medium, and high SDI groups.

### Statistical analyses

We calculated the estimated annual percentage change (EAPC) to assess the temporal trends of ASMR and ASDR over the past 30 years. The equation was set as follows: Y = α + βX + ε. In this linear regression model, X represents the calendar year, Y refers to the natural logarithm of ASDR or ASMR, ε indicates the error term. EAPCs were evaluated as 100 × (exp(β) – 1). We also calculated relevant 95% confidence interval (CI) by linear regression model mentioned earlier [15]. If the 95% CI is below 0, it suggests a declining trend. Conversely, if the 95% CI is above 0, it indicates an upward trend in the ASR. A stable trend is indicated when the 95% CI encompasses zero. Pearson's correlation analysis was conducted to examine the association between the EAPC estimates and SDI in the year 2019. R program (Version 4.12) was used for statistical analysis and visualization.

## Results

### Global breast cancer burden attributable to tobacco

**Fig. 1:**
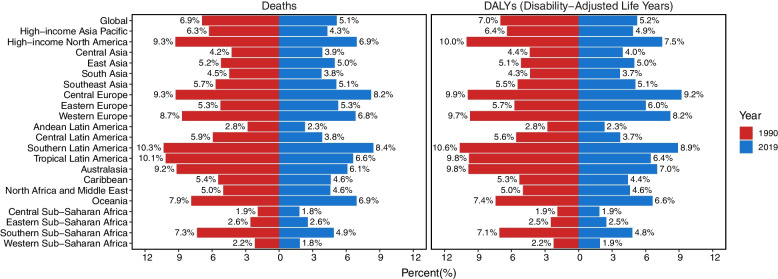
The percentage of breast cancer mortality and DALYs ascribed to tobacco, both on a global scale and within the 21 Global Burden of Disease (GBD) regions in 1990 and 2019

**Fig. 2:**
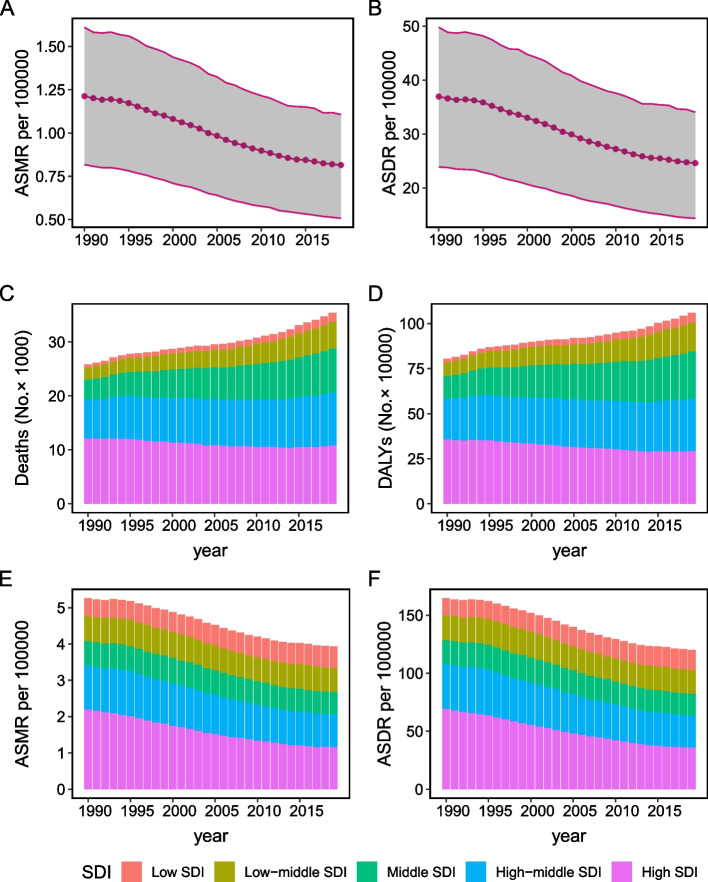
Change of tobacco-related breast cancer ASMR (**A**) and ASDR (**B**) during 1990–2019. The count of breast cancer deaths (**C**) and DALYs (**D**) linked to tobacco usage categorized by SDI levels between 1990 and 2019. ASMR (**E**) and ASDR (**F**) of breast cancer linked to tobacco usage categorized by SDI levels between 1990 and 2019. ASMR, age-standardized mortality rate; ASDR, age-standardized DALY rate; DALYs, disability-adjusted life-years

Out of the total breast cancer burden, the proportion of deaths and DALYs attributable to tobacco were estimated to be 5.1% and 5.2% respectively in 2019 (Fig. 1). The number of breast cancer deaths associated with tobacco witnessed an approximate increase of 35% during the past three decades, rising from 25,857 (95% UI: 17,283–34,333) to 35,439 (95% UI: 22,179–48,119) (Table 1). Concurrently, there was a significant increase in the corresponding DALYs, with a surge of approximately 30%, escalating from 805,990 (95% UI: 515,630–1,087,750) to 1,060,590 (95% UI: 622,550–1,462,580). By contrast, ASMR and ASDR were recorded as 0.82 (95% UI: 0.51–1.11) and 24.60 (95% UI: 14.37–34.04) per 100,000 population in 2019, indicating a declining trend, as evidenced by the negative EAPC (-1.55 for ASMR; -1.59 for ASDR) (Table 1, Fig. 2A and B). The deaths and DALYs in the regions with low, low-middle and middle SDI experienced an annual increase (Fig. 2C and D), while the relevant ASMR and ASDR in high-middle and high SDI regions exhibited a continuous decrease from 1990 to 2019 (Fig. 2E and F). The percentage of deaths and DALYs among these patients aged younger than 40 showed a slight decrease over the 30-year period. Conversely, there was a slight increase in these figures for individuals aged 70 and above (Figure S1).

**Table 1:** The global burden of breast cancer attributable to tobacco in 1990 and 2019 and the temporal trends during 1990–2019

Characteristics	1990				2019				EAPC of ASMR No. (95% CI)	EAPC of ASDR No. (95% CI)
	Death number No. × 10^2^ (95% UI)	ASMR per 100000 No. (95% UI)	DALY No. × 10^3^ (95% UI)	ASDR per 100000 No. (95% UI)	Death number No. × 10^2^ (95% UI)	ASMR per 100000 No. (95% UI)	DALY No. × 10^3^ (95% UI)	ASDR per 100000 No. (95% UI)		
Global	258.57 (172.83–343.33)	1.21 (0.82–1.61)	805.99 (515.63–1087.75)	36.93 (23.87–49.78)	354.39 (221.79–481.19)	0.82 (0.51–1.11)	1060.59 (622.55–1462.58)	24.60 (14.37–34.04)	-1.55 (-1.62–1.49)	-1.59 (-1.68–1.53)
Socio-demographic index										
Low SDI	6.16 (2.85–9.52)	0.49 (0.23–0.75)	20.85 (9.00–32.79)	14.66 (6.55–22.80)	16.69 (8.07–25.56)	0.59 (0.30–0.89)	55.09 (24.51–86.73)	17.24 (8.17–26.68)	0.62 (0.55–0.68)	0.49 (0.42–0.57)
Low-middle SDI	22.26 (10.33–33.99)	0.70 (0.34–1.05)	75.24 (32.30–116.39)	21.27 (9.56–32.65)	49.60 (21.97–76.16)	0.67 (0.31–1.03)	159.87 (65.09–249.53)	20.45 (8.58–31.80)	-0.3 (-0.4–0.2)	-0.34 (-0.46–0.22)
Middle SDI	37.55 (16.92–57.03)	0.67 (0.31–1.02)	127.68 (53.73–199.10)	20.95 (9.10–32.30)	81.26 (36.86–124.57)	0.61 (0.28–0.93)	261.27 (108.17–406.04)	18.88 (7.87–29.26)	-0.46 (-0.56–0.37)	-0.49 (-0.58–0.40)
High-middle SDI	70.97 (45.11–95.20)	1.20 (0.76–1.61)	226.20 (139.47–306.12)	38.56 (23.75–52.31)	98.71 (63.72–134.37)	0.91 (0.58–1.24)	291.51 (180.74–398.52)	27.92 (17.04–38.29)	-1.24 (-1.36–1.14)	-1.4 (-1.53–1.30)
High SDI	121.46 (88.12–152.26)	2.20 (1.61–2.75)	355.52 (257.28–446.58)	69.17 (50.15–87.03)	107.86 (77.52–137.29)	1.16 (0.83–1.46)	292.01 (210.18–368.17)	35.70 (25.54–45.15)	-2.4 (-2.51–2.35)	-2.46 (-2.56–2.43)
GBD region										
High-income Asia Pacific	5.97 (3.78–8.04)	0.54 (0.34–0.73)	20.38 (12.27–27.94)	18.70 (11.18–25.76)	8.82 (5.75–11.92)	0.47 (0.30–0.63)	25.99 (16.59–35.35)	16.39 (10.04–22.46)	-0.27 (-0.42–0.13)	-0.27 (-0.44–0.10)
High-income North America	48.65 (34.08–62.26)	2.69 (1.92–3.41)	145.93 (105.35–184.63)	86.93 (63.48–108.87)	42.21 (29.11–55.27)	1.31 (0.93–1.70)	115.43 (83.14–148.34)	39.67 (28.91–50.59)	-2.67 (-2.80–2.61)	-2.92 (-3.06–2.87)
Central Asia	2.21 (0.95–3.39)	0.80 (0.34–1.22)	7.45 (3.11–11.51)	26.84 (11.21–41.65)	2.91 (1.22–4.52)	0.65 (0.27–1.00)	9.73 (3.98–15.14)	20.38 (8.41–31.66)	-0.65 (-0.73–0.57)	-0.98 (-1.07–0.91)
East Asia	22.45 (7.99–36.68)	0.48 (0.18–0.77)	76.64 (25.23–128.11)	15.31 (5.13–25.44)	48.80 (21.21–78.60)	0.45 (0.20–0.73)	150.58 (58.84–244.45)	13.95 (5.37–22.62)	-0.38 (-0.50–0.27)	-0.56 (-0.68–0.44)
South Asia	17.60 (7.21–28.54)	0.60 (0.26–0.97)	59.99 (22.45–97.40)	17.98 (7.18–29.04)	47.44 (19.60–76.30)	0.64 (0.27–1.02)	153.47 (58.39–252.32)	19.40 (7.58–31.68)	-0.03 (-0.16–0.11)	0.03 (-0.12–0.18)
Southeast Asia	16.40 (6.91–25.37)	1.10 (0.49–1.68)	57.45 (21.89–92.89)	34.88 (14.21–55.13)	34.00 (13.90–54.54)	0.98 (0.41–1.56)	114.96 (42.79–189.75)	31.49 (11.89–51.81)	-0.5 (-0.61–0.40)	-0.45 (-0.54–0.36)
Central Europe	16.11 (11.49–20.56)	2.00 (1.43–2.58)	49.69 (35.34–64.01)	63.78 (45.15–82.91)	18.97 (13.25–25.42)	1.72 (1.21–2.31)	51.20 (36.22–69.11)	51.51 (35.96–69.72)	-0.67 (-0.74–0.60)	-0.91 (-0.99–0.83)
Eastern Europe	15.62 (8.21–22.42)	0.97 (0.50–1.39)	51.84 (26.71–74.45)	34.00 (17.69–48.93)	18.48 (10.85–26.60)	0.98 (0.57–1.41)	57.40 (33.23–82.80)	32.92 (18.92–47.72)	-0.57 (-0.82–0.33)	-0.76 (-1.02–0.50)
Western Europe	76.72 (56.24–95.61)	2.62 (1.92–3.29)	220.56 (160.96–277.57)	83.08 (60.56–105.03)	66.54 (48.63–84.20)	1.54 (1.12–1.93)	177.65 (129.05–223.65)	47.83 (34.36–60.79)	-2.12 (-2.26–2.03)	-2.17 (-2.29–2.10)
Andean Latin America	0.40 (0.20–0.60)	0.36 (0.18–0.53)	1.31 (0.61–1.99)	10.96 (5.27–16.45)	0.87 (0.46–1.33)	0.29 (0.16–0.44)	2.62 (1.28–4.12)	8.62 (4.29–13.47)	-1.01 (-1.16–0.88)	-1.16 (-1.31–1.02)
Central Latin America	3.41 (2.08–4.73)	0.75 (0.47–1.03)	10.75 (6.17–15.14)	21.80 (12.93–30.50)	6.41 (3.67–9.43)	0.50 (0.29–0.73)	19.25 (10.21–29.07)	14.58 (7.80–22.04)	-1.52 (-1.62–1.44)	-1.51 (-1.62–1.43)
Southern Latin America	7.40 (5.35–9.56)	2.93 (2.10–3.79)	21.05 (14.84–27.57)	84.24 (59.13–110.49)	9.39 (6.64–12.16)	2.07 (1.46–2.68)	24.47 (17.12–31.86)	57.03 (39.17–74.81)	-1.24 (-1.33–1.17)	-1.37 (-1.45–1.31)
Tropical Latin America	9.07 (6.60–11.62)	1.80 (1.31–2.30)	28.49 (20.07–36.74)	52.60 (37.46–67.64)	13.44 (9.45–17.56)	1.00 (0.70–1.31)	38.98 (26.72–51.81)	28.88 (19.80–38.37)	-2.09 (-2.42–1.81)	-2.11 (-2.46–1.80)
Australasia	2.98 (2.22–3.73)	2.50 (1.85–3.13)	9.09 (6.68–11.53)	79.87 (58.59–101.26)	2.71 (1.96–3.43)	1.14 (0.82–1.45)	7.96 (5.66–10.20)	36.74 (25.93–47.39)	-2.88 (-3.10–2.74)	-2.83 (-3.03–2.73)
Caribbean	1.47 (1.01–1.95)	1.09 (0.75–1.45)	4.45 (2.92–5.97)	32.25 (21.37–43.17)	2.63 (1.78–3.56)	0.96 (0.65–1.29)	7.34 (4.82–10.12)	27.14 (17.75–37.43)	-0.26 (-0.36–0.16)	-0.43 (-0.53–0.33)
North Africa and Middle East	5.75 (2.69–8.76)	0.61 (0.29–0.93)	20.28 (9.17–31.19)	19.83 (9.09–30.34)	16.17 (7.40–24.87)	0.69 (0.32–1.04)	56.14 (24.72–88.41)	21.76 (9.83–33.89)	0.46 (0.39–0.52)	0.33 (0.27–0.39)
Oceania	0.45 (0.22–0.66)	2.64 (1.37–3.89)	1.58 (0.75–2.39)	84.59 (41.62–126.51)	1.25 (0.62–1.99)	3.03 (1.57–4.71)	4.50 (2.09–7.31)	97.48 (47.91–155.93)	0.59 (0.53–0.64)	0.61 (0.55–0.67)
Central Sub-Saharan Africa	0.43 (0.19–0.70)	0.33 (0.15–0.52)	1.48 (0.60–2.46)	9.79 (4.12–16.12)	1.23 (0.50–2.12)	0.38 (0.16–0.65)	4.24 (1.62–7.37)	11.49 (4.64–19.88)	0.43 (0.30–0.55)	0.44 (0.32–0.55)
Eastern Sub-Saharan Africa	1.59 (0.70–2.47)	0.40 (0.19–0.61)	5.28 (2.14–8.43)	11.44 (4.98–17.91)	4.19 (2.02–6.47)	0.47 (0.25–0.71)	13.43 (5.84–21.56)	12.84 (5.97–19.91)	0.55 (0.49–0.60)	0.34 (0.26–0.42)
Southern Sub-Saharan Africa	2.16 (1.28–3.09)	1.37 (0.82–1.98)	6.64 (3.78–9.50)	39.04 (22.67–55.92)	3.47 (1.93–4.93)	1.07 (0.60–1.51)	9.91 (5.19–14.45)	28.49 (15.10–41.35)	-0.67 (-0.89–0.46)	-0.8 (-1.00–0.61)
Western Sub-Saharan Africa	1.71 (0.68–2.83)	0.38 (0.15–0.61)	5.65 (2.09–9.48)	11.38 (4.34–18.90)	4.48 (1.74–7.37)	0.41 (0.17–0.65)	15.36 (5.56–25.48)	12.07 (4.64–19.95)	0.24 (0.18–0.30)	0.2 (0.12–0.27)

*ASMR* Age-standardized mortality rate, *UI* Uncertainty interval, *DALYs* Disability-adjusted life years, *ASDR* Age-standardized DALY rate, *EAPC* Estimated annual percentage change, *CI* Confidence interval, *SDI* Socio-demographic Index

### Regional breast cancer burden attributable to tobacco

At the SDI-regional level, both the number of deaths and DALYs across five SDI regions in 2019 exceeded the corresponding values recorded in 1990 (Table 1). Quantitatively, there was a noticeable increase in the number of deaths and DALYs, as well as an increase in ASMR and ASDR, with the progressive rise in regional SDI in 2019. A noticeable decline in both ASMR and ASDR was observed in regions with high and high-middle SDI, with an EAPC of less than -1. By contrast, in low-middle and middle SDI regions, the improvement in ASMR and ASDR was less notable, as indicated by the EAPC approaching zero. It was important that low SDI region had a slight increase of ASMR (EAPC: 0.62, 95% UI: 0.55–0.68) and ASDR (EAPC: 0.49, 95% UI: 0.42–0.57).

In 2019, the GBD regions with the highest estimated death and DALYs due to tobacco-related breast cancer were Western Europe, recording 6,654 deaths and 17,7650 DALYs, East Asia with 4,880 deaths and 150,580 DALYs, and South Asia with 4,744 deaths and 153,470 DALYs (Table 1). Oceania was estimated to have the highest ASMR (3.03 per 100,000) and ASDR (97.48 per 100,000) attributed to tobacco-related breast cancer, followed closely by Southern Latin America (2.07 per 100,000 for ASMR, 57.03 per 100,000 for ASDR) and Central Europe (1.72 per 100,000 for ASMR, 51.51 per 100,000 for ASDR). From 1990 to 2019, ASMR and ASDR of tobacco-related breast cancer exhibited a downward trend in most regions worldwide. However, a slight upward trend of ASMR and ASDR with a positive EAPC was observed in North Africa and Middle East, Oceania, Central Sub-Saharan Africa, Eastern Sub-Saharan Africa, and Western Sub-Saharan Africa.

Based on Fig. 1, the death and DALYs proportion attributable to tobacco exhibited a similar regional distribution in 1990 and 2019. The contribution of tobacco to breast cancer death and DALYs declined in all GBD regions from 1990 to 2019, except for Eastern Europe and Eastern Sub-Saharan Africa.

### Countries and territories trends of breast cancer attributable to tobacco

**Fig. 3:**
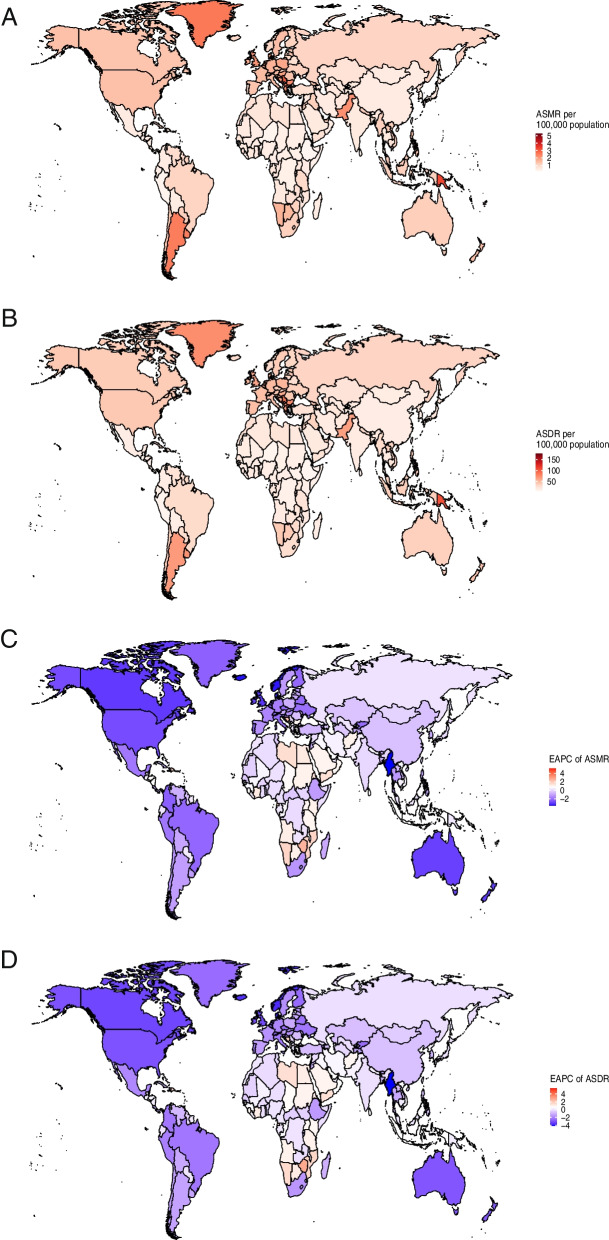
Tobacco-related breast cancer ASMR (**A**) and ASDR (**B**), and their corresponding EAPC (**C** and **D**) across 204 countries and territories in 2019. ASMR, age-standardized mortality rate; ASDR, age-standardized DALY rate; EAPC, estimated annual percentage change

At the national level, the burden of tobacco-related breast cancer varied significantly across 204 countries or territories in 2019 (Table S1). An upward trend in ASMR was documented in 88 countries or territories, a downward trend was observed in 95 countries or territories, and a stable status was recorded in 21 countries or territories. Furthermore, we noted an increase in ASDR in 76 countries or territories, a decrease in ASDR in 96 countries or territories, and a stable ASDR in the remaining 32 countries or territories. China had the highest number of deaths (4,671, 95% UI: 2,024–7,572) and DALYs (143,660, 95% UI: 56,490–233,810), followed by United States of America and India (Tables S2 and S3). The highest estimated national-level ASMR and ASDR in 2019 were documented in specific countries within Oceania (such as Solomon Islands, Nauru, Tuvalu, and Papua New Guinea) as well as Central Europe (including Montenegro and Serbia) (Fig. 3A and B, Tables S4 and S5). Solomon Islands, Lesotho, and Zimbabwe displayed the highest EAPC of ASMR and ASDR. Conversely, Israel, Denmark, Norway, Iceland, and Israel had the lowest EAPC of ASMR and ASDR, with the values lower than -3. (Fig. 3C and D, Tables S6 and S7).

### Global breast cancer burden attributable to tobacco by age, SDI, and HDI

**Fig. 4:**
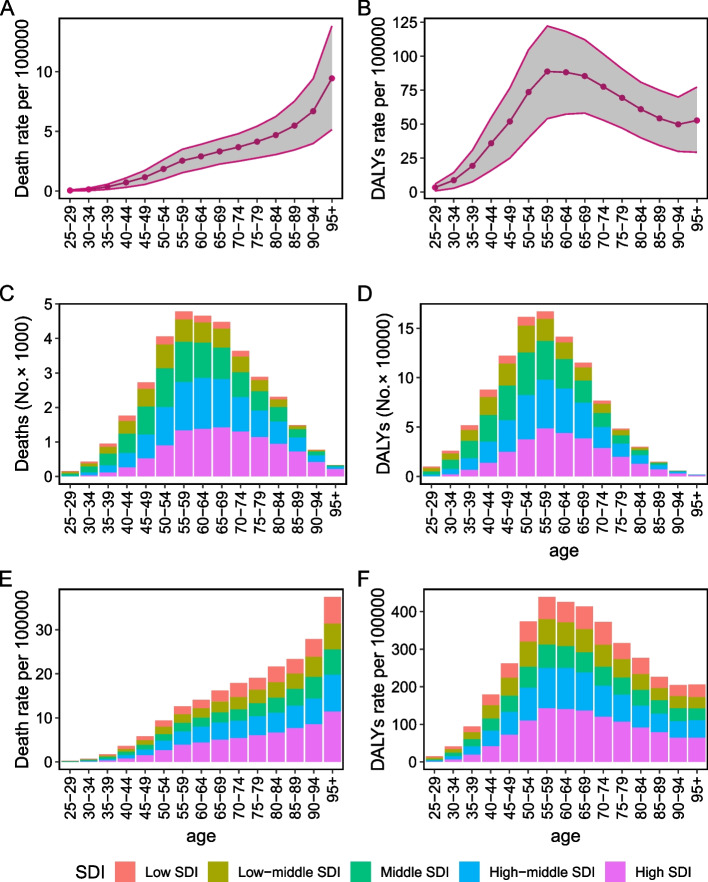
The distribution of deaths (**A**) and DALYs (**B**) rate attributed to tobacco-related breast cancer by age. Breast cancer deaths (number, **C**; rate, **D**) and DALYs (number, **E**; rate, **F**) attributable to tobacco by SDI and age. The shaded area represents the 95% uncertainty interval for the rate. DALYs, disability-adjusted life-years; SDI, socio-demographic index

The death rate of tobacco-related breast cancer exhibited a gradual global increase in correlation with advancing age in 2019 (Fig. 4A). The highest rate of DALYs occurred in the individuals aged 55–59 (Fig. 4B). A similar pattern was evident in Fig. 4E and F, with approximately half of the deaths and DALYs occurring in the High-middle SDI and High SDI regions. Corresponding, there was a notable increase in the number of deaths and DALYs as age advanced, reaching their highest point within the 55–59 age group (Fig. 4C and D).

The age-specific death rate experienced an obvious decrease in High SDI region, particularly among individuals aged 45–90. It declined slightly in Low-middle SDI, Middle SDI, and High-middle SDI regions, while showing a moderate increase in Low SDI region (Figure S2A). Irrespective of SDI, the mortality rate increased among patients aged above 95. The patterns observed in the EAPCs of age-specific DALY rates mirrored those of age-specific mortality rates (Figure S2B).

**Fig. 5:**
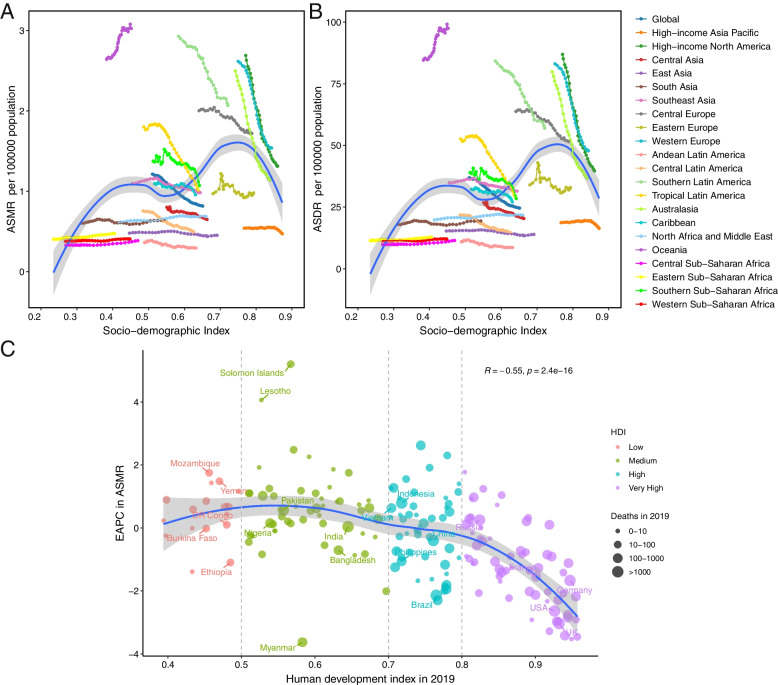
The relationship between breast cancer burden attributable to tobacco and socio-demographic index among 21 Global Burden of Disease regions. **A** Age-standardized death rate breast cancer attributed to tobacco; **B** Age-standardized DALY rate of breast cancer attributed to tobacco. **C** The association between EAPC in age-standardized mortality rate and HDI in 2019. ASMR, age-standardized death rate; ASDR, age-standardized DALYs rate; DALYs, disability-adjusted life-years, EAPC, estimated annual percentage change; HDI, human development index

A double hump curve was depicted to illustrate the relationship between SDI and the regional ASMR or ASDR (Fig. 5A and B). Among the 21 GBD regions, the observed patterns in ASRs varied widely based on the year. Some regions showed minimal change in ASRs, while others exhibited decreasing or fluctuating rates. Additionally, North Africa and Middle East experienced a progressive rise in ASMR and ASDR over time. There was a positive relationship between ASRs and SDI value in 2019 across 204 nations or territories (R = 0.29, *p* < 0.01 for both ASMR and ASDR, Figure S3). However, a negative association was observed between the EAPC in ASMR and the HDI in 2019 (R = -0.55, *p *< 0.01), which was particularly pronounced in nations with very high HDI, but not in nations with HDI less than 0.8 (Fig. 5C). We also observed a comparable association between EAPC in ASDR and HDI in 2019 (Figure S4).

## Discussion

This study represents the most current and comprehensive investigation examining the spatial and temporal trends of tobacco-associated breast cancer on a global scale. Our analysis unveiled that the global ASMR and ASDR of tobacco-related breast cancer decreased modestly over the last three decades, but the death and DALYs cases increased by about 30%, which may be partially attributed to population aging and growth [16]. In parallel, we observed both death and DALYs number were highest in the 55–59 age group. Moreover, the spatial distribution of this disease was heterogeneous, implying a potential correlation with disparities in various factors, including breast cancer screening policies and tobacco control initiatives.

Tobacco smoke is recognized to contain more than 7,000 chemicals, with about 20 of them being identified as breast carcinogens [17]. They may have an impact on the development, recurrence, metastasis, and treatment of breast cancer through various potential biological mechanisms. Firstly, nicotine, the principal component of tobacco, contributes to breast cancer by promoting proliferation, inhibiting apoptosis, and creating a microenvironment conducive to tumor growth [18]. Secondly, tobacco smoke can increase the risk of metastasis from breast cancer, which is attributed mainly to phenotypic transition to a mesenchymal phenotype, acquisition of self-renewing stem-like traits, chronic inflammation, and inhibition of host immune defenses [19, 20]. Thirdly, several population-based studies revealed that tobacco increased both breast-specific mortality and overall mortality [21, 22]. In comparison to men, women displayed a higher susceptibility to genetic and biologic aberrations caused by tobacco, thereby increasing their vulnerability to cancer development [23–25]. Women exposed to second-hand smoke in both the workplace and home had a significantly higher risk of developing breast cancer [26]. The absence of second-hand smoke exposure in women who have never smoked was suggested to potentially prevent 1 out of every 14 cases of breast cancer [27]. In addition, the cessation of smoking following disease diagnosis significantly improved breast cancer specific survival and overall survival [28]. However, the cessation rate after breast cancer diagnosis was lower compared to other cancers such as colorectal and lung cancer [29–31]. It is imperative to urgently propose feasible policy measures for comprehensive smoke control worldwide, particularly in regions grappling with a substantial disease burden of tobacco-related breast cancer.

We demonstrated that the burden of breast cancer attributable to tobacco varied notably among regions and nations in this study. Australasia, High-income North America, West Europe, and Tropical Latin America experienced a substantial reduction in both ASMR and ASDR. These declines may be partly ascribed to the widespread implementation of mammogram screening programs and evidence-based tobacco control policies in these regions [32, 33]. On the contrary, our findings indicated a slight upward trend of ASMR and ASDR in North Africa and Middle East, Oceania, Central Sub-Saharan Africa, and Eastern Sub-Saharan Africa. Both ASMR and ASDR increased in Low SDI region, but not in other SDI regions. These frustrating trends could be explained by factors such as limited availability of treatment, inadequate implementation of health policies, and challenging living conditions [34–36]. The enforcement of cigarette advertisement bans, tobacco tax policies, and warning labels has been correlated with a reduction in tobacco consumption and a decrease in overall cancer mortality [33, 37, 38]. Hence, recommendations for screening initiation ages can be tailored to specific risk factors, including national risk profiles and ethnicity [39]. The governments should concentrate their efforts on levying higher taxes on tobacco products and deploying smoke-free legislation, individuals should strive earnestly to quit smoke and cultivate healthy habits in the countries and regions with high tobacco-related breast cancer burden.

In our analysis, we conducted comprehensive estimations of the disease burden and trends associated with tobacco-related female breast cancer at global, regional, and national levels based on multiple metrics, including mortality, DALYs, ASMR, and ASDR. Nevertheless, it is important to acknowledge and address several limitations. First, variations in data collection methods and mammogram screening practices among different nations inevitably impact the dependability of our findings. Second, molecular subtyping and grade are significant clinicopathological parameters for breast cancer. Given the scarcity of data, we did not include these characteristics in our analysis. Third, the GBD 2019 study offers valuable and reliable estimates of disease burden, yet the data regarding the prevalence and incidence of tobacco-related breast cancer are unavailable. Fourth, Pham et al. indicated that electronic cigarettes can potentially facilitate the growth and metastasis of breast cancer [40]. Regrettably, our study lacks exposure estimates for the use of electronic cigarettes, vaporizers, and heated tobacco products due to inadequate data availability in the GBD 2019 dataset.

In conclusion, tobacco is one important and modifiable risk factor for breast cancer. A number of regions (namely North Africa and Middle East, and Oceania) and countries or territories (namely Solomon Islands, Lesotho, and Zimbabwe) exhibited a notable upward trajectory. In addition, the populations in Low SDI regions would suffer a substantial burden of tobacco-related breast cancer, leading to a persistent challenge to both healthcare and economic sectors in the foreseeable future. These findings substantiate the necessity of expediting the enforcement of tobacco-free legislation in order to safeguard populations from the adverse effects of tobacco.

### Supplementary Information

**Additional file 1: Figure S1. **The proportion of different age groups in breast cancer attributed to tobacco during 30 years. 

**Additional file 2: Figure S2. **The EAPC in death rate (A) and DALYs rate (B) of tobacco-related breast cancer over a 30-year period by age and SDI. EAPC, estimated annual percentage change; DALYs, disability-adjusted life-years; SDI, sociodemographic index.

**Additional file 3: Figure S3. **The relationship between tobacco-related breast cancer burden and socio-demographic index in 2019. Age-standardized morality rate (A) and Age-standardized DALYs rate (B). The blue line represents an adaptive association obtained by fitting adaptive Loess regression using all available data points. DALYs, disability-adjusted life-years.

**Additional file 4: Figure S4.** The association between EAPC in age-standardized DALYs rate and HDI in 2019. EAPC, estimated annual percentage change; DALYs, disability-adjusted life-years, HDI, human development index.

**Additional file 5: Table S1. **The burden of breast cancer attributable to tobacco across 204 nations or regions in 1990 and 2019 and the temporal trends during 1990-2019.

**Additional file 6: Table S2. **Top 10 nations or regions exhibiting the highest number of breast cancer mortalities associated with tobacco in 2019.

**Additional file 7: Table S3. **Top 10 nations or regions exhibiting the highest number of breast cancer DALYs associated with tobacco in 2019.

**Additional file 8: Table S4. **Top 10 countries or territories with the highest breast cancer ASMR (per 100000) attributable to tobacco in 2019.

**Additional file 9: Table S5. **Top 10 countries or territories with the highest breast cancer ASDR (per 100000) attributable to tobacco in 2019.

**Additional file 10: Table S6. **Top 10 nations or regions with the highest or lowest EAPC in breast cancer ASMR (per 100000) attributable to tobacco from 1990 to 2019.

**Additional file 11: Table S7.** Top 10 nations or regions with the highest or lowest EAPC in breast cancer ASDR (per 100000) attributable to tobacco from 1990 to 2019.

## Data Availability

Publicly available datasets were analyzed in this study. The data can be found here: http://ghdx.healthdata.org/gbd-results-tool and http://hdr.undp.org/en/data.

## References

[1] Makhoul I, Atiq M, Alwbari A, Kieber-Emmons T (2018). Breast Cancer Immunotherapy: An Update. Breast Cancer.

[2] Ferlay J, Colombet M, Soerjomataram I, Parkin DM, Pineros M, Znaor A, Bray F (2021). Cancer statistics for the year 2020: An overview. Int J Cancer..

[3] Giaquinto AN, Sung H, Miller KD, Kramer JL, Newman LA, Minihan A, Jemal A, Siegel RL (2022). Breast Cancer Statistics, 2022. CACancer J Clin..

[4] Sun YS, Zhao Z, Yang ZN, Xu F, Lu HJ, Zhu ZY, Shi W, Jiang J, Yao PP, Zhu HP (2017). Risk Factors and Preventions of Breast Cancer. Int J Biol Sci.

[5] Collaborators GBDRF: Global burden of 87 risk factors in 204 countries and territories, 1990–2019: a systematic analysis for the Global Burden of Disease Study 2019. Lancet. 2020;396(10258):1223–49. 10.1016/S0140-6736(20)30752-2 PMC7566194 33069327

[6] Goodchild M, Nargis N, Tursan d'Espaignet E (2018). Global economic cost of smoking-attributable diseases. Tob Control.

[7] Hecht SS, Hatsukami DK (2022). Smokeless tobacco and cigarette smoking: chemical mechanisms and cancer prevention. Nat Rev Cancer.

[8] Jones ME, Schoemaker MJ, Wright LB, Ashworth A, Swerdlow AJ (2017). Smoking and risk of breast cancer in the Generations Study cohort. Breast Cancer Res.

[9] Li N, Deng Y, Zhou L, Tian T, Yang S, Wu Y, Zheng Y, Zhai Z, Hao Q, Song D (2019). Global burden of breast cancer and attributable risk factors in 195 countries and territories, from 1990 to 2017: results from the Global Burden of Disease Study 2017. J Hematol Oncol.

[10] Ji P, Gong Y, Jin ML, Hu X, Di GH, Shao ZM (2020). The Burden and trends of breast cancer from 1990 to 2017 at the global, regional, and national levels: results from the global burden of disease study 2017. Front Oncol.

[11] Scala M, Bosetti C, Bagnardi V, Possenti I, Specchia C, Gallus S, Lugo A (2023). Dose-Response Relationships between Cigarette Smoking and Breast Cancer Risk: A Systematic Review and Meta-Analysis. JEpidemiol..

[12] He Y, Si Y, Li X, Hong J, Yu C, He N (2022). The relationship between tobacco and breast cancer incidence: A systematic review and meta-analysis of observational studies. Front Oncol.

[13] Diseases GBD, Injuries C (2020). Global burden of 369 diseases and injuries in 204 countries and territories, 1990–2019: a systematic analysis for the Global Burden of Disease Study 2019. Lancet.

[14] Collaborators GBDRF: Global, regional, and national comparative risk assessment of 84 behavioural, environmental and occupational, and metabolic risks or clusters of risks for 195 countries and territories, 1990–2017: a systematic analysis for the Global Burden of Disease Study 2017. Lancet. 2018;392(10159):1923–94. 10.1016/S0140-6736(18)32225-6 PMC6227755 30496105

[15] Lu Y, Lan T (2022). Global, regional, and national burden of hypertensive heart disease during 1990–2019: an analysis of the global burden of disease study 2019. BMC Public Health.

[16] Arnold M, Morgan E, Rumgay H, Mafra A, Singh D, Laversanne M, Vignat J, Gralow JR, Cardoso F, Siesling S (2022). Current and future burden of breast cancer: Global statistics for 2020 and 2040. Breast.

[17] Reynolds P (2013). Smoking and breast cancer. J Mammary Gland Biol Neoplasia.

[18] Khodabandeh Z, Valilo M, Velaei K, Pirpour Tazehkand A (2022). The potential role of nicotine in breast cancer initiation, development, angiogenesis, invasion, metastasis, and resistance to therapy. Breast Cancer.

[19] Di Cello F, Flowers VL, Li H, Vecchio-Pagan B, Gordon B, Harbom K, Shin J, Beaty R, Wang W, Brayton C (2013). Cigarette smoke induces epithelial to mesenchymal transition and increases the metastatic ability of breast cancer cells. Mol Cancer.

[20] Liu Y, Gu Y, Han Y, Zhang Q, Jiang Z, Zhang X, Huang B, Xu X, Zheng J, Cao X (2016). Tumor Exosomal RNAs Promote Lung Pre-metastatic Niche Formation by Activating Alveolar Epithelial TLR3 to Recruit Neutrophils. Cancer Cell.

[21] Wang K, Li F, Zhang X, Li Z, Li H (2016). Smoking increases risks of all-cause and breast cancer specific mortality in breast cancer individuals: a dose-response meta-analysis of prospective cohort studies involving 39725 breast cancer cases. Oncotarget.

[22] Passarelli MN, Newcomb PA, Hampton JM, Trentham-Dietz A, Titus LJ, Egan KM, Baron JA, Willett WC (2016). Cigarette smoking before and after breast cancer diagnosis: mortality from breast cancer and smoking-related diseases. J Clin Oncol.

[23] Jemal A, Miller KD, Ma J, Siegel RL, Fedewa SA, Islami F, Devesa SS, Thun MJ (2018). Higher lung cancer incidence in young women than young men in the United States. N Engl J Med.

[24] Barrera-Rodriguez R, Morales-Fuentes J (2012). Lung cancer in women. Lung Cancer (Auckl).

[25] Alisoltani A, Qiu X, Jaroszewski L, Sedova M, Iyer M, Godzik A (2023). Gender differences in smoking-induced changes in the tumor immune microenvironment. Arch Biochem Biophys.

[26] Carreras G, Lugo A, Gallus S, Cortini B, Fernandez E, Lopez MJ, Soriano JB, Lopez-Nicolas A, Semple S, Gorini G (2019). Burden of disease attributable to second-hand smoke exposure: A systematic review. Prev Med.

[27] Gram IT, Wiik AB, Lund E, Licaj I, Braaten T (2022). Never-smokers and the fraction of breast cancer attributable to second-hand smoke from parents during childhood: the Norwegian Women and Cancer Study 1991–2018. Int J Epidemiol.

[28] Singareeka Raghavendra A, Kypriotakis G, Karam-Hage M, Kim S, Jizzini M, Seoudy KS, Robinson JD, Barcenas CH, Cinciripini PM, Tripathy D (2022). The Impact of Treatment for Smoking on Breast Cancer Patients' Survival. Cancers..

[29] Steinhilper L, Geyer S, Sperlich S (2013). Health behavior change among breast cancer patients. Int J Public Health.

[30] Hopenhayn C, Christian WJ, Christian A, Studts J, Mullet T (2013). Factors associated with smoking abstinence after diagnosis of early stage lung cancer. Lung Cancer.

[31] Skeie G, Hjartaker A, Braaten T, Lund E (2009). Dietary change among breast and colorectal cancer survivors and cancer-free women in the Norwegian Women and Cancer cohort study. Cancer Causes Control.

[32] Siu AL, Force USPST: Screening for Breast Cancer: U.S. Preventive Services Task Force Recommendation Statement. Ann Int Med. 2016;164(4):279–96. 10.7326/M15-2886 26757170

[33] Jiang H, Livingston M, Room R, Gan Y, English D, Chenhall R (2019). Can public health policies on alcohol and tobacco reduce a cancer epidemic? Australia's experience. BMC Med.

[34] Balogun O, Rodin D, Ngwa W, Grover S, Longo J (2017). Challenges and prospects for providing radiation oncology services in Africa. Sem Radiation Oncol.

[35] Jemal A, Center MM, DeSantis C, Ward EM (2010). Global patterns of cancer incidence and mortality rates and trends. Cancer Epidemiol Biomarkers Prev.

[36] Chowdhury S, Pillarisetti A, Oberholzer A, Jetter J, Mitchell J, Cappuccilli E, Aamaas B, Aunan K, Pozzer A, Alexander D (2023). A global review of the state of the evidence of household air pollution's contribution to ambient fine particulate matter and their related health impacts. Environ Int.

[37] Levy DT, Tam J, Kuo C, Fong GT, Chaloupka F (2018). The Impact of Implementing Tobacco Control Policies: The 2017 Tobacco Control Policy Scorecard. J Public Health Manage Pract.

[38] Hoffman SJ, Tan C (2015). Overview of systematic reviews on the health-related effects of government tobacco control policies. BMC Public Health.

[39] Chen T, Kharazmi E, Fallah M (2023). Race and ethnicity-adjusted age recommendation for initiating breast cancer screening. JAMA Netw Open.

[40] Pham K, Huynh D, Le L, Delitto D, Yang L, Huang J, Kang Y, Steinberg MB, Li J, Zhang L (2020). E-cigarette promotes breast carcinoma progression and lung metastasis: Macrophage-tumor cells crosstalk and the role of CCL5 and VCAM-1. Cancer Lett.

