# Supplementary figures and images for: The global, regional, and national disease burden of breast cancer attributable to tobacco from 1990 to 2019: a global burden of disease study
Source: BMC Public Health. 2024 Jan 6;24:107. doi: 10.1186/s12889-023-17405-w (PMC10770986; doi:10.1186/s12889-023-17405-w)

A

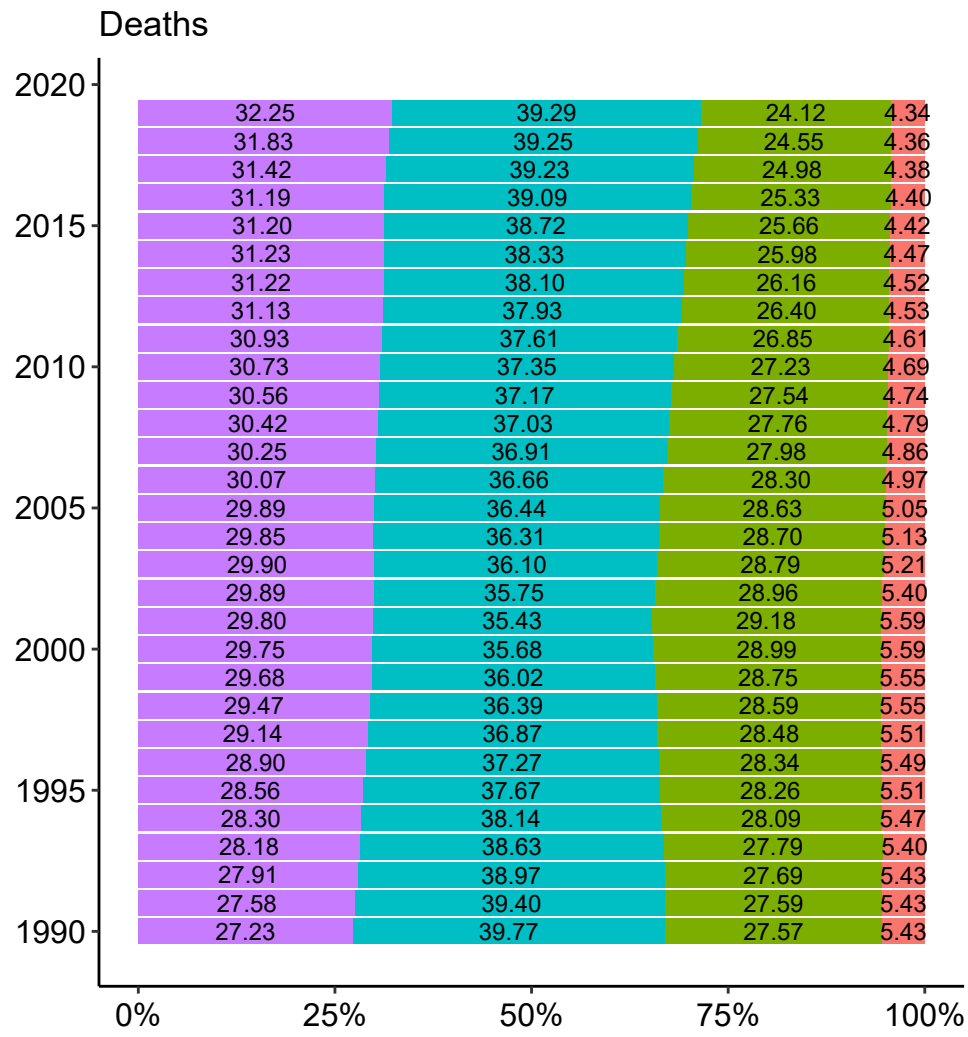

B

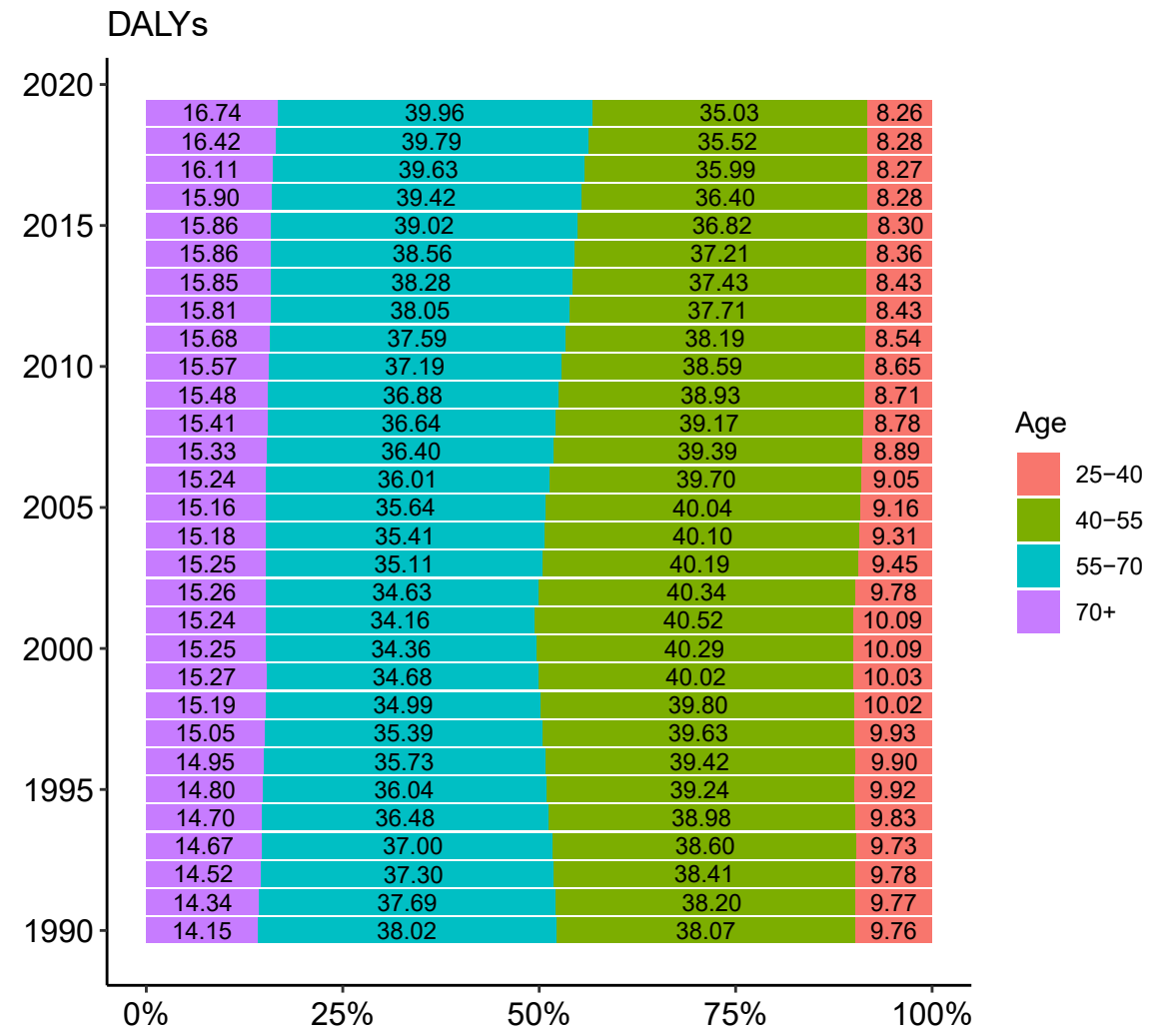

Age

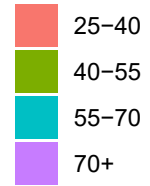

Supplement: Supplementary file 1 — Additional file 1: Figure S1. The proportion of different age groups in breast cancer attributed to tobacco during 30 years. [file 12889_2023_17405_MOESM1_ESM.pdf]

A

## EAPC in death rate

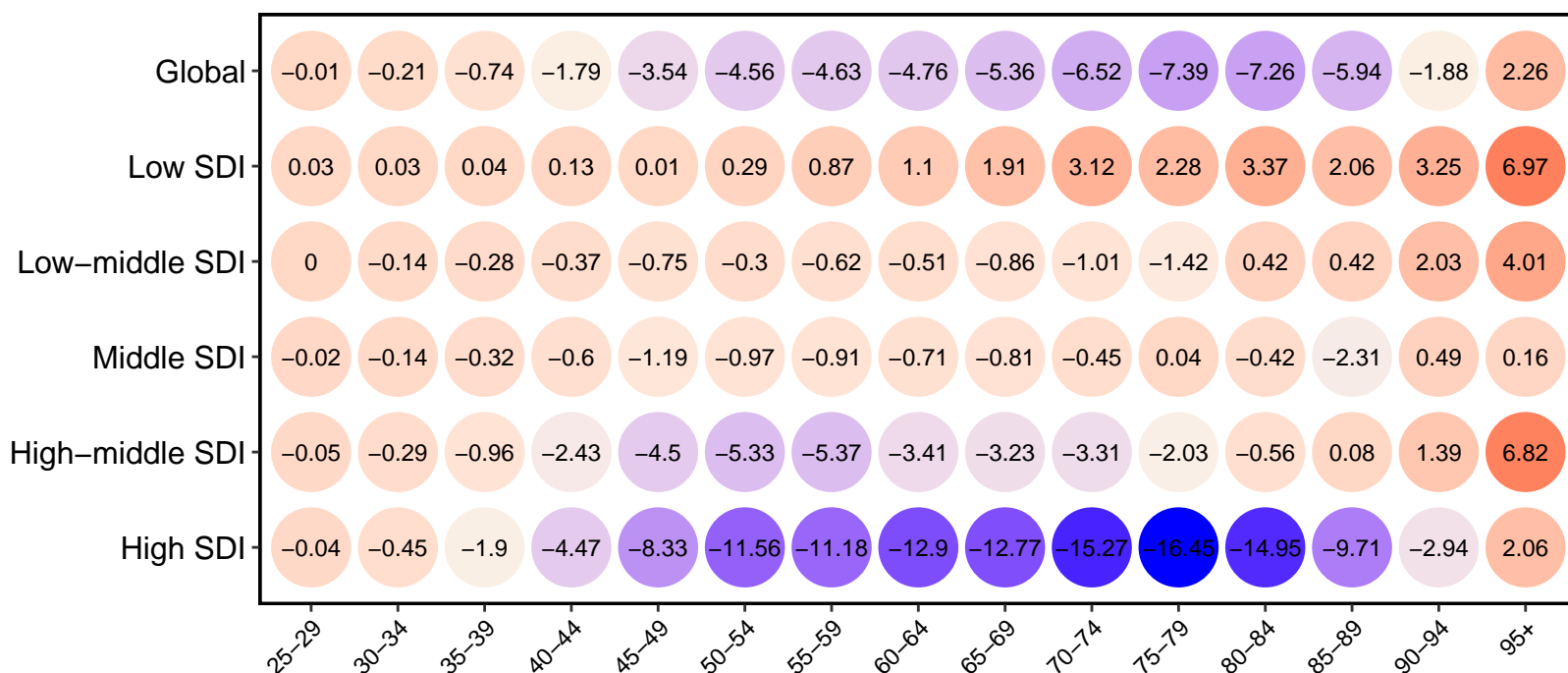

B

## EAPC in DALYs rate

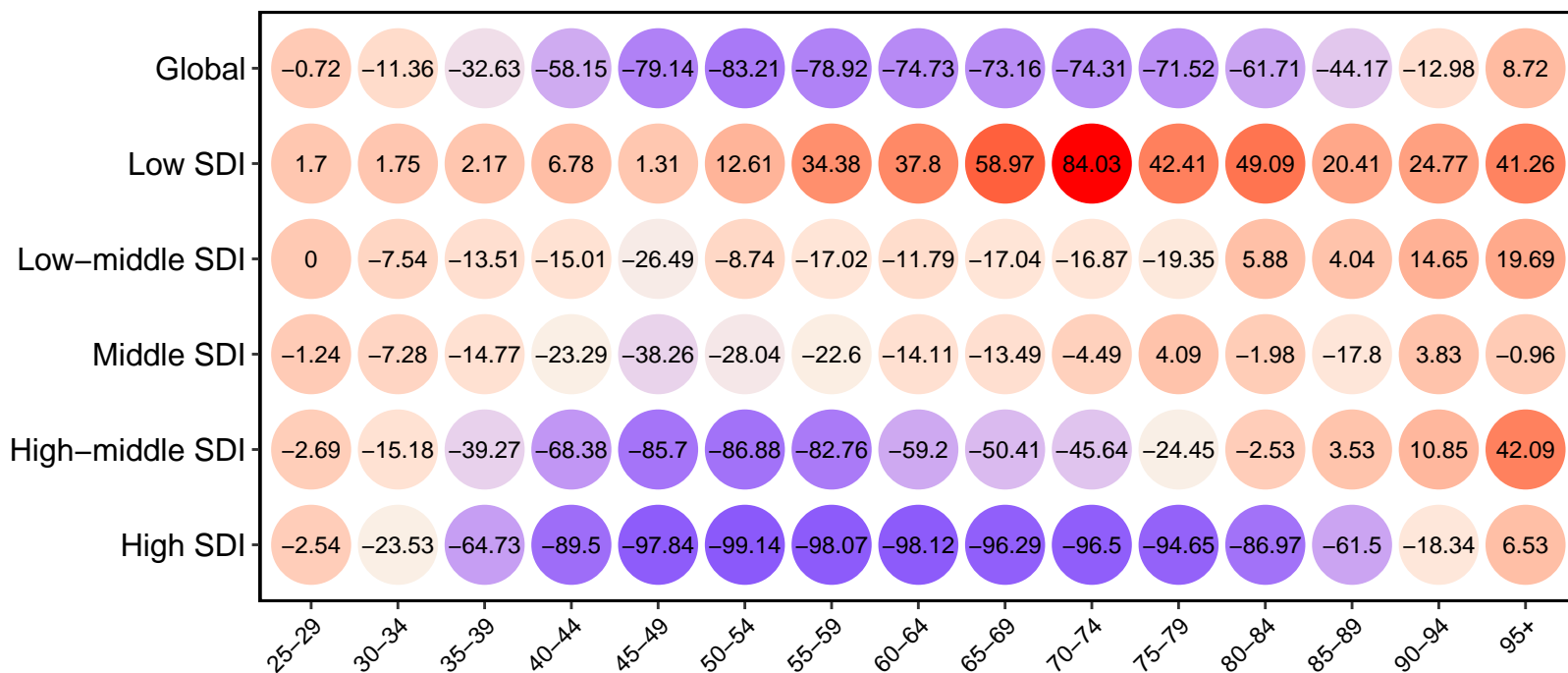

Supplement: Supplementary file 2 — Additional file 2: Figure S2. The EAPC in death rate (A) and DALYs rate (B) of tobacco-related breast cancer over a 30-year period by age and SDI. EAPC, estimated annual percentage change; DALYs, disability-adjusted life-years; SDI, sociodemographic index. [file 12889_2023_17405_MOESM2_ESM.pdf]

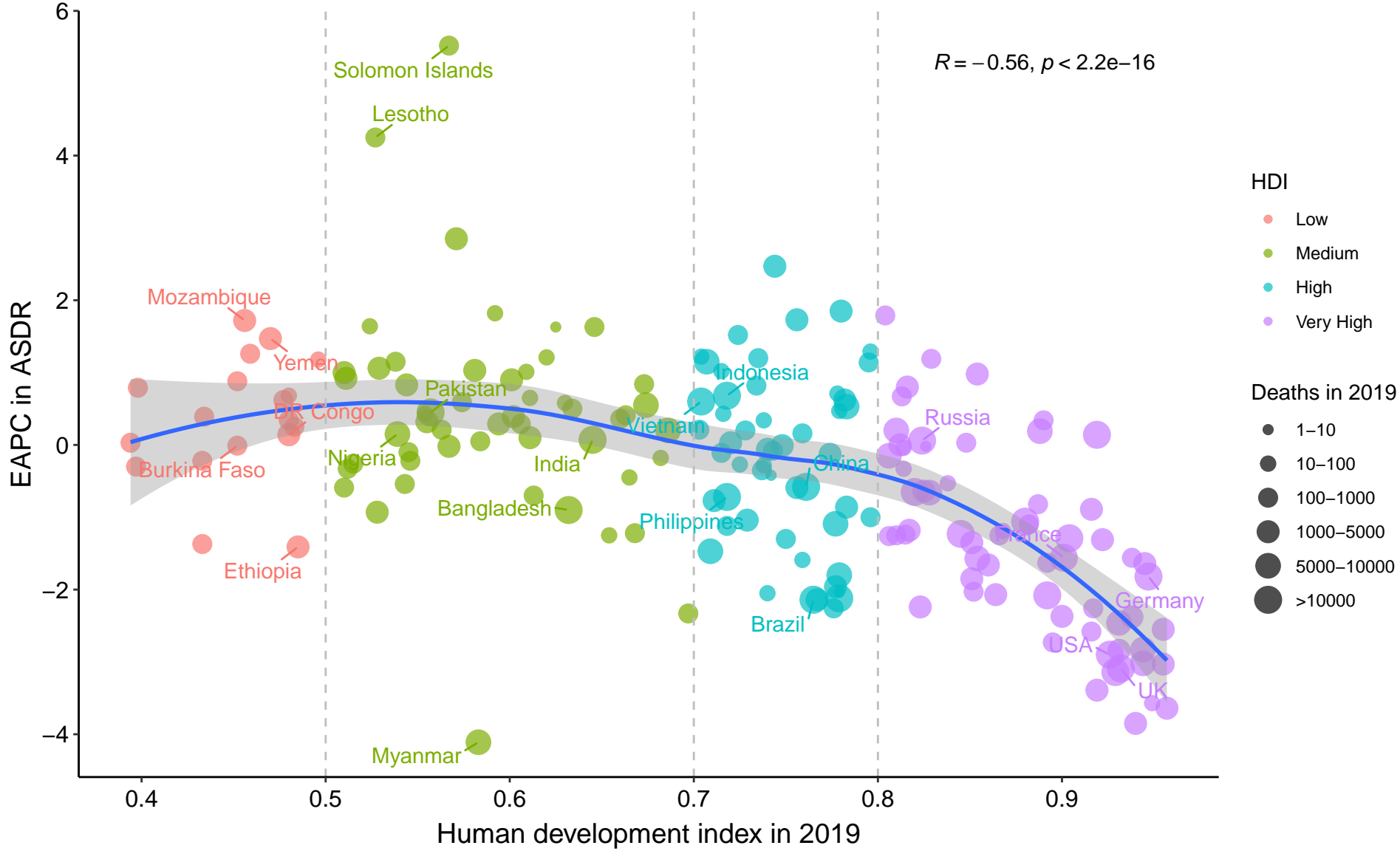

Supplement: Supplementary file 4 — Additional file 4: Figure S4. The association between EAPC in age-standardized DALYs rate and HDI in 2019. EAPC, estimated annual percentage change; DALYs, disability-adjusted life-years, HDI, human development index. [file 12889_2023_17405_MOESM4_ESM.pdf]
