# Supplementary material for: The global, regional, and national disease burden of breast cancer attributable to tobacco from 1990 to 2019: a global burden of disease study
Source: BMC Public Health. 2024 Jan 6;24:107. doi: 10.1186/s12889-023-17405-w (PMC10770986; doi:10.1186/s12889-023-17405-w)
Supplement: Supplementary file 3 — Additional file 3: Figure S3. The relationship between tobacco-related breast cancer burden and socio-demographic index in 2019. Age-standardized morality rate (A) and Age-standardized DALYs rate (B). The blue line represents an adaptive association obtained by fitting adaptive Loess regression using all available data points. DALYs, disability-adjusted life-years. [file 12889_2023_17405_MOESM3_ESM.pdf]

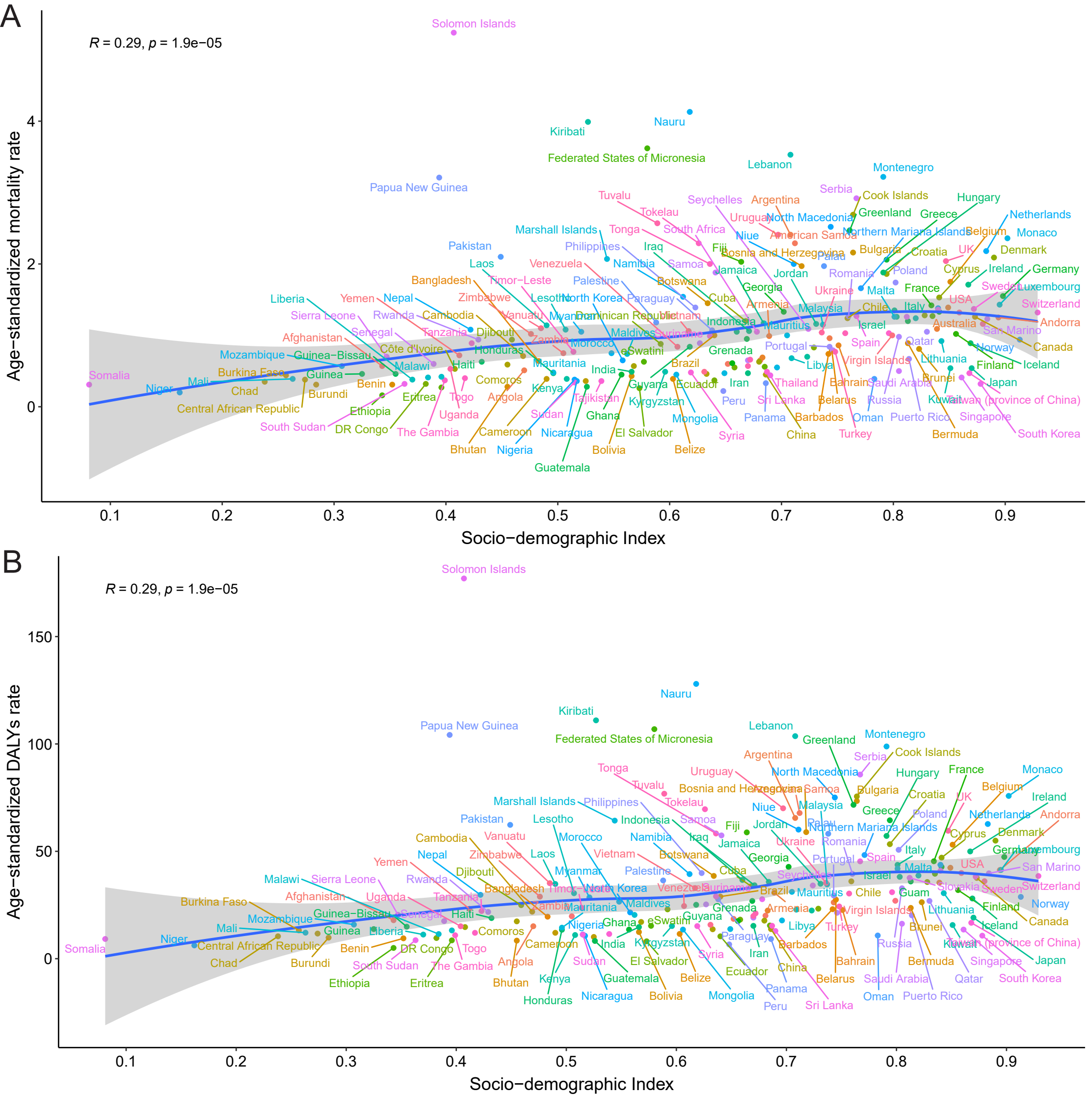
A
R = 0.29, p = 1.9e−05
Age−standardized mortality rate
Socio−demographic Index
0
2
4
0.1
0.2
0.3
0.4
0.5
0.6
0.7
0.8
0.9
Solomon Islands
Nauru
Kiribati
Federated States of Micronesia
Lebanon
Montenegro
Papua New Guinea
Serbia
Cook Islands
Hungary
Tuvalu
Seychelles
Argentina
Greenland
Greece
Netherlands
Tokelau
Marshall Islands
Tonga
South Africa
Monaco
Pakistan
Philippines
Iraq
Denmark
Laos
Venezuela
Namibia
Samoa
Jamaica
Jordan
Romania
Poland
Cyprus
Ireland
Germany
Bangladesh
Timor−Leste
Palestine
Botswana
Georgia
Ukraine
Malta
France
Luxembourg
Liberia
Yemen
Nepal
Zimbabwe
North Korea
Paraguay
Cuba
Armenia
Malaysia
Chile
Italy
Switzerland
Sierra Leone
Rwanda
Cambodia
Vanuatu
Myanmar
Dominican Republic
Vietnam
Indonesia
Mauritius
Israel
Australia
Andorra
Senegal
Tanzania
Djibouti
Zambia
Maldives
Suriname
Portugal
Spain
Qatar
San Marino
Afghanistan
Côte d'Ivoire
Morocco
Norway
Canada
Mozambique
Guinea−Bissau
Honduras
eSwatini
Grenada
Virgin Islands
Lithuania
Iceland
Malawi
Haiti
Mauritania
Brazil
Libya
Finland
Somalia
Burkina Faso
Guinea
India
Brunei
Japan
Mali
Comoros
Niger
Benin
Kenya
Guyana
Ecuador
Iran
Thailand
Bahrain
Saudi Arabia
Chad
Burundi
Togo
Angola
Tajikistan
Kyrgyzstan
Peru
Sri Lanka
Belarus
Russia
Kuwait
Taiwan (province of China)
Central African Republic
Eritrea
Ethiopia
Uganda
Sudan
Ghana
Mongolia
Panama
Barbados
Oman
Puerto Rico
Singapore
South Sudan
DR Congo
The Gambia
Cameroon
Nicaragua
El Salvador
Syria
China
Turkey
Bermuda
South Korea
Bhutan
Nigeria
Bolivia
Belize
Guatemala
B
R = 0.29, p = 1.9e−05
Age−standardized DALYs rate
Socio−demographic Index
0
50
100
150
0.1
0.2
0.3
0.4
0.5
0.6
0.7
0.8
0.9
Solomon Islands
Nauru
Kiribati
Papua New Guinea
Federated States of Micronesia
Lebanon
Greenland
Montenegro
Argentina
Serbia
Cook Islands
Tonga
Uruguay
North Macedonia
Hungary
France
Monaco
Tuvalu
Bulgaria
Belgium
Tokelau
Croatia
UK
Ireland
Marshall Islands
Philippines
Malaysia
Greece
Netherlands
Andorra
Pakistan
Lesotho
Indonesia
Niue
Jordan
Samoa
Poland
Fiji
Northern Mariana Islands
Cyprus
Cambodia
Vanuatu
Morocco
Namibia
Iraq
Jamaica
Ukraine
Romania
Denmark
Laos
Vietnam
Botswana
Georgia
Italy
Nepal
Zimbabwe
Portugal
Spain
Yemen
Djibouti
Myanmar
Palestine
Cuba
Malta
USA
San Marino
Malawi
Sierra Leone
Rwanda
Seychelles
Israel
Bangladesh
North Korea
Suriname
Mauritius
Chile
Guam
Switzerland
Afghanistan
Uganda
Tanzania
Slovakia
Sweden
Burkina Faso
Maldives
Norway
Guinea−Bissau
Senegal
Haiti
Zambia
Mauritania
Armenia
Virgin Islands
Lithuania
Finland
Mozambique
Ghana
eSwatini
Guyana
Canada
Mali
Guinea
Liberia
Comoros
Nigeria
Libya
Turkey
Brunei
Iceland
Niger
Cameroon
India
Kyrgyzstan
Paraguay
Iran
Barbados
Russia
Kuwait
Taiwan (province of China)
Somalia
Central African Republic
Benin
DR Congo
Togo
Japan
Chad
Burundi
South Sudan
The Gambia
Angola
Sudan
El Salvador
Syria
China
Bahrain
Bermuda
Singapore
Ecuador
Belize
Guatemala
Belarus
Saudi Arabia
Qatar
South Korea
Ethiopia
Eritrea
Bhutan
Kenya
Panama
Oman
Puerto Rico
Honduras
Nicaragua
Bolivia
Mongolia
Peru
Sri Lanka
